# Clinicopathological and prognostic significance of FOXP3+ tumor infiltrating lymphocytes in patients with breast cancer: a meta-analysis

**DOI:** 10.1186/s12885-015-1742-7

**Published:** 2015-10-17

**Authors:** Daqing Jiang, Zhaohua Gao, Zhengang Cai, Meixian Wang, Jianjun He

**Affiliations:** Department of Breast Surgery, Liaoning Province Cancer Hospital and Institute, Shenyang, Liaoning 110042 People’s Republic of China; Department of Surgical Oncology, the First Affiliated Hospital, School of Medicine of Xi’an Jiaotong University, Xi’an, 710061 Shaanxi Province China; Department of Breast Surgery, Dalian Medical University Clinical Oncology College, Shenyang, Liaoning 110042 China; Department of Breast Surgery, the First Affiliated Hospital, Dalian Medical University, Dalian, Liaoning 116011 China; Department of Tumour Pathology and Surgical Oncology, First Hospital of China Medical University, Shenyang, 110001 China

**Keywords:** Breast cancer, Regulatory T cell, FOXP3, Tumor-infiltrating lymphocytes, Prognosis, Meta-analysis

## Abstract

**Background:**

The prognostic significance of FOXP3+ tumor-infiltrating lymphocytes (TILs) in patients with breast cancer remains controversial. The aims of our meta-analysis are to evaluate its association with clinicopathological characteristics and prognostic significance in patients with breast cancer.

**Methods:**

PubMed, Embase, Cochrane Database and the Ovid Database were systematically searched (up to April 2015). The meta-analysis was performed using hazard ratio (HR), odds ratio (OR) and 95 % confidence intervals (CI) as effect measures. Using the random-effects model, statistical analysis was performed using Stata software, version 12.0.

**Results:**

Seventeen studies including 8277 patients with breast cancer were analyzed. The meta-analysis indicated that the incidence difference of FOXP3+ TILs was significant when comparing the lymph node positive group to negative group (OR = 1.305, 95 % CI [1.071, 1.590]), the histological grade III group to the I–II group (OR = 3.067, 95 % CI [2.288, 4.111]), the ER positive group to the negative group (OR = 0.435, 95 % CI [0.287, 0.660]), the PR positive group to the negative group (OR = 0.493, 95 % CI [0.296, 0.822]), the HER2 positive group to the negative group (OR = 1.896, 95 % CI [1.335, 2.692]), the TNBC group to the non TNBC group (OR = 2.456, 95 % CI [1.801, 3.348]). The detection of FOXP3+ TILs was significantly correlated with the recurrence-free survival (RFS) of patients (HR = 1.752, 95 % CI [1.188–2.584]) and the overall survival (OS) of patients (HR =1.447, 95 % CI [1.037–2.019]).

**Conclusions:**

Our meta-analysis demonstrates that the presence of high levels of FOXP3+ TILs is associated with prognosis for breast cancer patients and predicts lymph node metastasis, hormone receptor and HER-2 status.

## Background

Breast cancer is the most common type of diagnosed cancer in women [1] and is still the second leading cause of cancer-related death among women all over the world [2]. So far, prediction of outcome is still not optimal and additional predictive and prognostic factors are needed to improve individualized treatment. A large number of evidence has proved the existence of immune surveillance function disorders against tumor cells in breast cancer patients [3, 4]. Tumor may shape the local immune microenvironment by recruiting lymphocytes, which regulate and release inflammatory mediators with pro-angiogenic or pro-metastatic effects [5]. In the tumor microenvironment, complex network of immune suppression influence the effects of anticancer treatments,and the accumulation of regulatory T cell indicates an important working model of the network. The investigations of tumor microenvironment can reveal the complex relationship between the tumor cell biology and immune system. In order to control breast cancer, a deep understanding of tumor microenvironment will prove to be very important.

In the process of tumorigenesis, tumor progression and metastatic spread, effective evasion of the immune system by tumor cells is essential. The type, density and location of tumor-infiltrating lymphocytes (TILs) within the tumor have shown to be predictors of survival rate in breast cancer patients [6–8]. Regulatory T cells (Treg cells) is considered to be involved in the maintenance of immune tolerance, prevent autoimmune diseases and suppress antitumor immune responses. More and more evidence indicates that regulatory T cells play an important role in immune evasion mechanisms of cancer [9–12]. Tumor actively recruit and induce regulatory T cells to prevent innate and adaptive immunity starts, effector function and memory response, which may lead to a favorable environment for the development of cancer. Forkhead box protein 3 (FOXP3) is a member of the forkhead/winged-helix family of transcription factors involved in regulating immune system development and function [13, 14]. This gene plays a important role in the generation of immunosuppressive CD4 + CD25+ regulatory T cells (Tregs), which induce immune tolerance to antigens [14, 15]. Loss of FOXP3 function leads to a lack of Tregs, resulting in lethal autoaggressive lymphoproliferation, whereas FOXP3 overexpression results in severe immunodeficiency [14, 15]. FOXP3 has been considered the most specific marker for Treg cells [16, 17]. Tumor-induced FOXP3 + regulatory T cells increasing indicates a potential barrier to attempts at cancer immunotherapy. Cancer patients may benefit from blocking the capacity of tumor cells to recruit Tregs. To date, the prognostic significance of FOXP3+ TILs in breast cancer remains controversial. However, a meta-analysis demonstrating an association between FOXP3+ TILs detection and prognosis has not yet been performed.

The aims of our study were to use meta-analysis to demonstrate the correlation between FOXP3+ TILs and the clinicopathological characteristics of breast cancer and evaluate whether detection of FOXP3+ TILs can act as a clinical predictor for patients with breast cancer.

## Methods

### Search strategy

PubMed, Embase, Cochrane Database and the Ovid Database were systematically searched for studies addressing the clinicopathological and prognostic correlation between FOXP3+ TILs and breast cancer without language, place of publication or time restrictions (up to April 2015). No search restrictions were applied. Furthermore, the reference lists of the retrieved studies and reviews were reviewed for further identification of potential relevant articles. The main search terms used were “FOXP3+”, “TILs”, “Tumor-infiltrating lymphocytes”, “prognosis”, “Regulatory T cell”, “breast cancer”, “breast carcinoma”.

### Inclusion criteria

To ensure that our analysis is accurate and reliable, eligible studies were selected based on the following criteria: (i) The prognostic or clinicopathological significance of FOXP3+ TILs detection in breast cancer patients with at least one of the interested outcome measures was reported in the study or can be calculated from published data. (ii) Immunohistochemistry (IHC) detection methods was used to detect specific FOXP3 antigens with monoclonal anti-human FOXP3. (iii) Samples were collected from the core-needle biopsy or postoperative surgery specimens. Reporting hazard ratio (HR), odds ratio (OR) and their 95 % confidence interval (CI); if not, the reported data of outcomes RFS, OS and pCR were sufficient to calculate them.

Two reviewers (Z.H. Gao and D.Q. Jiang) independently carried out literature searches and determined eligible articles based on the inclusion criteria. Disagreements between reviewers were resolved via discussion and consensus. If they can not reach agreement, a third researcher to determine the final results (J. He). If multiple publications were based on the same patient population, we utilized the most informative study.

### Data extraction and quality assessment

We extracted our data based on Cochrane guidelines [18]. Two reviewers (Z.H. Gao, D.Q. Jiang) reviewed eligible studies independently, and any disagreements were resolved through discussion and consensus. Extracted information for this meta-analysis included: first author, publication years, the journal, trial design, baseline patient characteristics, age range, dosing regimens, patient eligibility, clinicopathological characteristics, follow-up period, TILs site, cut-off point, end-points (RFS, OS, pCR) , hazard ratio (HR) and 95 % confidence interval (CI). The quality of the included studies was evaluated according to the Newcastle-Ottawa scale (NOS) criteria for cohort studies [19] Publication bias was assessed by funnel plot. The written informed consents of all participants have been described and obtained by all the original studies that were included in our meta-analysis. The original studies were conducted in accordance with all local regulations, Good Clinical Practice principles and the Declaration of Helsinki.

### Statistical analysis

The prognostic effect of the meta-analysis were recurrence-free survival (RFS), overall survival (OS). Effect measures regarding the effect in the meta-analysis were reported as hazard ratio (HR) with 95 % confidence interval (CI). The estimated odds ratio (OR) was used to summarize the correlation between FOXP3+ TILs detection and breast cancer clinicopathological characteristics. If the HR and its 95 % confidence interval (95 % CI) were not reported directly in the original study, they were calculated from the available data using software designed by Tierney et al. [20] Heterogeneity among studies was calculated using the Q test and I^2^ value indicates the degree of heterogeneity [21]. I^2^ of <40 % indicates low heterogeneity [18]. If outcomes with low heterogeneity, a fixed-effect model was used; otherwise random effects models were used. The P value threshold for statistical significance was set at 0.05 for effect sizes. The overall analysis was performed by assessing all the relevant researches according to different clinicopathological parameters and prognostic outcomes. Meanwhile, subgroup analysis was completed in each clinicopathological parameter on the basis of the different TILs site and different countries. A sensitivity analysis was performed to evaluate the quality and consistency of results. Publication bias was tested using the funnel plot.

Statistical analysis was performed using Stata software, version 12.0 (2011) (Stata Corp, College Station, TX, USA). The recommendations of the Preferred Reporting Items for Systematic Reviews and Meta-analyses (PRISMA) was utilized as a guideline for this meta-analysis [22].

### Ethics statement

The study was conducted in accordance with the local regulations, and was approved by the Ethics Committee of the Liaoning Province Cancer Hospital and Institute.

## Results

### Characteristics of the eligible studies

**Fig. 1:**
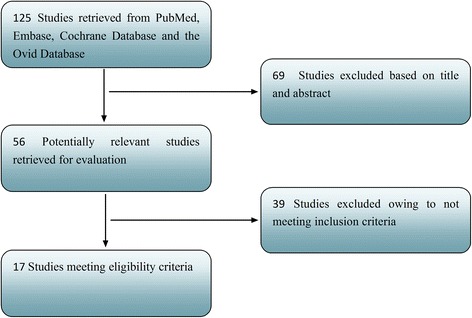
Flow chart of studies selection

We identified a total of 125 studies in systematic literature search. 56 potentially relevant studies were identified by reviewing the titles and abstracts. In the remaining 56 studies, 39 studies were then excluded because they do not meet the selection criteria. Finally, the remaining 17 studies met the selection criteria and included in the meta-analysis [7, 23–38]. The searching and screening procedure is summarized in Fig. 1. The 17 studies included 8277 eligible breast cancer patients (sample size median: 153 [72–3277], mean: 487). The studies were from Asia, Europe and North America (Japan, Korea, China, France, United Kingdom, Netherlands and Canada) and were published between 2006 and 2014. All the included studies detected FOXP3+ TILs with IHC method. In terms of the evaluation of FOXP3+ TILs site, one study evaluated the significance of FOXP3+ TILs detection at intratumoural, peritumoral and distant stromal separately, [27] five studies evaluated the significance of FOXP3+ TILs at intratumoural and peritumoral separately, [7, 28, 33, 36, 37] three evaluated the significance of FOXP3+ TILs detection only at intratumoural, [25, 34, 38] two studies assessed the significance of FOXP3+ TILs detection only at peritumoral [26, 30] and six studies evaluated the significance of FOXP3+ TILs detection did not distinguish sites (total sites) [23, 24, 29, 31, 32, 35]. Sixteen studies provided HRs on RFS or OS to complete the meta-analysis. Eight of the 16 studies were available for HRs on OS, [7, 23, 27, 28, 30, 31, 33, 34] and eight studies were available for HRs on RFS [7, 23, 25, 28–30, 32, 33, 35] The main baseline characteristics is summarized in Table 1. The quality of the included studies was assessed according to the NOS and is summarized in Table 2.

**Table 1:** Baseline characteristics of included trials

First author	Publication years	Country	Number (n)	CRT	Age Median (range)	TILs subsets	Sample time	TILs site	Method	curative resection	Outcome measured	Follow up Median (range)(M)
Ladoire S et al.[26]	2011	France	162	YES/YES	NR	FOXP3+/CD8+	B-NC/post	Peritumoral	IHC	YES	RFS; OS	NR
Lee S et al. [30]	2013	Korea	86	NR/YES	NR	FOXP3+	NR	Peritumoral	IHC	NR	RFS;OS	73.5(24.2–120.0)
Mahmoud SMA et al. [27]	2011	United Kingdom	1445	NR/YES	NR	FOXP3+	Post	Intratumoural;distant stromal; peritumoral	IHC/TMA	YES	OS;RFS	128(4–243)
Liu F et al. [7]	2011	China	1270	NO/YES	52(19–92)	FOXP3+/CD8+	Post	Intratumoural; peritumoral	IHC	YES	OS; RFS	66(1–78)
West NR et al. [32]	2013	Canada	175	NR	NR	FOXP3+/CD8+	Post	Total	IHC	YES	RFS; OS	83
Bates GJ et al. [23]	2006	United Kingdom	299	NR/YES	NR	FOXP3+	Post	Total	IHC	YES	RFS; OS	87.6(2.4–135.6)
Takenaka M et al. [31]	2013	Japan	100	NO/NR	NR	FOXP3+	Post	Total	IHC	YES	OS; RFS	NR
Maeda N et al. [35]	2014	Japan	90	NO/YES	NR	FOXP3+	Post	Total	IHC	YES	OS; RFS	67(7.8–90.5)
Sun S et al. [36]	2014	China	208	NO/YES	57.6(31–85)	FOXP3+/CD8+/PD-1	Post	Intratumoural; peritumoral	IHC	YES	OS; RFS	72(8.04–102.24)
Aruga T et al. [24]	2009	Japan	87	YES/NR	51(23–69)	FOXP3+	B-NC	Total	IHC	NR	OS; RFS	46.3(5.3–89.1)
De Kruijf EM et al. [25]	2010	Netherlands	556	NO/YES	57(23–96)	HCA2/HC10/Foxp3+	Post	Intratumoural	IHC	YES	OS; RFS	228(0–276)
Liu F et al. [28]	2012	China	132	YES/YES	53(38–72)	FOXP3+	B-NC/post	Intratumoural; peritumoral	IHC	YES	pCR; OS; RFS	62(18–73)
Liu S et al. [34]	2014	Canada	3277	NR/YES	58.9(23–95)	FOXP3+/CD8+	Post	Intratumoural	IHC/TMA	NR	OS; RFS	151(1.2–222)
Kim ST et al. [29]	2013	Korea	72	YES/NR	49(16–83)	FOXP3+/CD8+/CD4+	B-NC/post	Total	IHC	YES	RFS	34(21.9–38.3)
Tsang JY et al. [37]	2014	China	84	NO/NR	56.3(44.4–68.2)	FOXP3+/CD8+	Post	Intratumoural; peritumoral	IHC/TMA	YES	NO	NR
Kim S et al. [33]	2014	Korea	143	NO/NR	NR	FOXP3+/CD8+/CD4+	Post	Intratumoural; peritumoral	IHC	YES	OS; RFS	69
Seo AN et al. [38]	2013	Korea	153	YES/NR	NR	FOXP3+/CD8+/CD4+	B-NC/post	Intratumoural	IHC	YES	pCR;	NR

*CRT*, chemoradiotherapy (pre/postoperation); *TILs* Tumor-infiltrating lymphocytes; *IHC* Immunohistochemistry; *TMA* tissue microarrays; *B-NC* before Neoadjuvant chemotherapy; *post* postoperative; *NR* Not reported; *RFS* recurrence-free survival; OS: overall survival; *pCR* pathologic complete response

**Table 2:** The assessment of the risk of bias in each cohort study using the Newcastle–Ottawa scale

Study	Selection (0–4)				Comparability (0–2)		Outcome (0–3)			Total
	REC	SNEC	AE	DO	SC	AF	AO	FU	AFU	
Ladoire S et al. [26]	1	1	1	1	0	0	1	0	0	5
Lee S et al. [30]	0	1	1	0	0	0	1	1	0	4
Mahmoud SMA et al. [27]	1	1	1	1	0	0	1	1	0	6
Liu F et al. [7]	1	1	1	1	1	0	1	1	0	7
West NR et al. [32]	1	1	1	1	0	0	1	1	0	6
Bates GJ et al. [23]	1	1	1	1	0	0	1	1	0	6
Takenaka M et al. [31]	1	1	1	1	0	0	1	1	0	6
Maeda N et al. [35]	1	1	1	1	0	0	1	1	0	6
Sun S et al. [36]	1	1	1	1	1	0	1	1	1	8
Aruga T et al. [24]	1	1	1	1	0	0	0	1	0	5
De Kruijf EM et al. [25]	1	1	1	1	1	0	1	1	0	7
Liu F et al. [28]	1	1	1	1	0	0	1	1	0	6
Liu S et al. [34]	1	1	1	1	0	0	1	1	0	6
Kim ST et al. [29]	0	1	1	1	0	0	0	0	0	3
Tsang JY et al. [37]	1	1	1	1	0	0	0	0	0	4
Kim S et al. [33]	1	1	1	1	0	0	0	1	0	5
Seo AN et al. [38]	1	1	1	1	0	0	0	0	0	4

*REC* representativeness of the exposed cohort; *SNEC* selection of the non exposed cohort; *AE* ascertainment of exposure; *DO* demonstration that outcome of interest was not present at start of study; *SC* study controls for age, sex; *AF* study controls for any additional factor; *AO* assessment of outcome; *FU* follow-up long enough for outcomes to occur (36 Months); *AFU* Adequacy of follow up of cohorts (≥90 %).“1” means that the study is meeted the item and “0” means the opposite situation

### Correlation of FOXP3+ TILs with clinicopathological parameters

#### The incidence of FOXP3+ TILs in the lymph node metastasis

The meta-analysis of all involved studies on lymph node metastasis showed a significantly higher incidence of FOXP3+ TILs in the lymph node positive group relative to the lymph node negative group (OR = 1.305, 95 % CI [1.071, 1.590], I^2^ = 60.0 %). Then subgroup analysis were performed on TILs site (Intratumoural: OR = 1.121, 95 % CI [0.953, 1.318], I^2^ = 38.4 %; Peritumoral: OR = 2.917, 95 % CI [1.067, 7.971], I^2^ = .%; Total: OR = 1.590, 95 % CI [1.057, 2.394], I^2^ = 65.9 %) and different countries (Asia: OR = 1.636, 95 % CI [0.993, 2.693], I^2^ = 71.2 %; Europe and North America: OR = 1.209, 95 % CI [1.017, 1.437], I^2^ = 41.6 %). The results of pooled analysis on breast cancer lymph node metastasis are summarized in Table 3.

**Table 3:** The detailed subgroup analysis results of clinicopathological parameters

Clinicopathological parameters	TILs site				Different countries	
	Any	Intratumoural	Peritumoral	Total	Asia	Europe/North America
Age > 50 vs. ≤ 50 (OR)	0.867[0.699,1.076]; I2 = 66.3 %; z = 1.30; *p* = 0.195	0.855[0.562,1.303]; I2 = 89.8 %; z = 0.73; *p* = 0.466	_	0.804[0.669,0.965 ]; I2 = 0.0 %; z = 2.35; *p* = 0.019	1.081[0.894,1.307 ]; I2 = 0.0 % ; z = 0.81 ; *p* = 0.420	0.731[0.592, 0.901]; I2 = 55.6 %; z = 2.93 ; *p* = 0.003
Tumour size > 2 cm vs. ≤ 2 cm (OR)	1.151[0.997,1.329]; I2 = 25.0 %; z = 1.92 ; *p* = 0.055	1.098[0.966,1.247]; I2 = 0.0 %; z = 1.43; *p* = 0.151	_	1.268[0.954,1.686 ]; I2 = 37.0 %; z = 1.64 ; *p* = 0.101	1.296[0.867,1.935 ]; I2 = 54.9 %; z = 1.26 ; *p* = 0.206	1.146[1.016 , 1.293]; I2 = 0.0 %; z = 2.22; *p* =0.026
LN(+) vs. LN(−)(OR)	1.305[1.071 ,1.590]; I2 = 60.0 %; z = 2.64; *p* =0.008	1.121[0.953 ,1.318]; I2 = 38.4 %; z = 1.37; *p* = 0.169	2.917[1.067 ,7.971]; I2 = .%; z = 2.09; *p* = 0.037	1.590[1.057 ,2.394]; I2 = 65.9 %; z = 2.22; *p* = 0.026	1.636[0.993 ,2.693]; I2 = 71.2 % ; z = 1.93; *p* =0.053	1.209[1.017 ,1.437 ]; I2 = 41.6 %; z = 2.16; *p* =0.031
pT:T3/T4 vs.T1/T2 (OR)	0.990[0.748 ,1.310]; I2 = 0.0 % ; z = 0.07; *p* =0.943	0.990[0.748 ,1.310]; I2 = 0.0 % ; z = 0.07; *p* = 0.943	_	_	_	0.990[0.748 ,1.310]; I2 = 0.0 % ; z = 0.07; *p* = 0.943
StageIII/IV vs.I/II (OR)	1.115[0.631 ,1.970 ]; I2 = 68.7 %; z = 0.37; *p* =0.709	0.925[0.642 ,1.335]; I2 = 30.7 %; z = 0.41; *p* = 0.679	_	_	1.181[0.418,3.341 ]; I2 = 79.1 %; z = 0.31; *p* =0.754	_
Histological grade:III vs.I(OR)	3.769[2.596, 5.472]; I2 = 64.6 %; z = 6.98; *p* < 0.0001	3.360[1.774, 6.363]; I2 = 79.0 %; z = 3.72; *p* < 0.0001	_	4.298[3.221, 5.736]; I2 = 0.0 %; z = 9.88; *p* < 0.0001	6.248[3.627,10.763]; I2 = .%; z = 6.60; *p* < 0.0001	3.342[2.270, 4.920]; I2 = 60.0 % ; z = 6.11; *p* < 0.0001
III vs. II (OR)	2.299[1.719,3.075]; I2 = 80.3 %; z = 5.61; *p* < 0.0001	1.945[1.551,2.439]; I2 = 56.9 %; z = 5.76; *p* < 0.0001	_	3.422[2.706,4.326]; I2 = 0.0 %; z = 10.29; *p* < 0.0001	2.287[1.740,3.005]; I2 = .%; z = 5.94; *p* < 0.0001	2.304[1.561, 3.400]; I2 = 85.2 % ; z = 4.20; *p* < 0.0001
II vs. I (OR)	1.596[1.172,2.174]; I2 = 51.3 %; z = 2.97; *p* = 0.003	1.790[1.191,2.691]; I2 = 51.2 %; z = 2.80; *p* = 0.005	_	1.260[0.947,1.677]; I2 = 18.7 %; z = 1.37; *p* = 0.171	2.732[1.636,4.562]; I2 = .%; z = 3.84; *p* < 0.0001	1.351 [1.087, 1.680]; I2 = 0.0 % ; z = 2.64 ; *p* = 0.008
Lymphatic invasion (+) vs.(−) (OR)	1.382[0.844,2.262]; I2 = 42.8 %; z = 1.29; *p* = 0.198	_	_	_	2.071[1.045,4.102]; I2 = 0.0 %; z = 2.09; *p* = 0.037	_
Vessel invasion (+) vs.(−) (OR)	1.107[0.750,1.634]; I2 = 24.0 %; z = 0.51; *p* = 0.608	_	_	1.107[0.750,1.634]; I2 = 24.0 %; z = 0.51; *p* = 0.608	_	_
ER (+) vs.(−) (OR)	0.435[0.287,0.660]; I2 = 90.3 %; z = 3.91; *p* < 0.0001	0.571[0.276,1.181]; I2 = 95.7 %; z = 1.51; *p* = 0.131	_	0.347[0.252,0.478]; I2 = 31.7 %; z = 6.48; *p* < 0.0001	0.419[0.193,0.908]; I2 = 88.8 %; z = 2.21; *p* = 0.027	0.481[0.324,0.714]; I2 = 84.8 %; z = 3.63; *p* < 0.0001
PR (+) vs.(−) (OR)	0.493[0.296,0.822]; I2 = 89.9 %; z = 2.71; *p* = 0.007	0.417[0.128,1.357]; I2 = 96.8 %; z = 1.45; *p* = 0.146	_	0.501[0.405,0.621]; I2 = 0.0 %; z = 6.31; *p* < 0.0001	0.432[0.195,0.959]; I2 = 85.1 %; z = 2.06; *p* = 0.039	0.594[0.373,0.945]; I2 = 79.2 %; z = 2.20; *p* = 0.028
HER2 (+) vs.(−) (OR)	1.896[1.335,2.692]; I2 = 75.1 %; z = 3.58; *p* < 0.0001	1.141[0.718,1.814]; I2 = 81.6 %; z = 0.56; *p* = 0.576	2.299[1.066,4.960]; I2 = .%; z = 2.12; *p* = 0.034	3.651[2.638,5.052]; I2 = 0.0 %; z = 7.81; *p* < 0.0001	1.684[0.881,3.218]; I2 = 74.4 %; z = 1.58; *p* = 0.115	2.059[1.203,3.523]; I2 = 81.0 %; z = 2.64; *p* = 0.008
Molecular Subtypes :TNBC vs. nTNBC (OR)	2.456[1.801,3.348]; I2 = 11.3 %; z = 5.68; *p* < 0.0001	3.514[1.563,7.901]; I2 = .%; z = 3.04; *p* = 0.002	_	2.342[1.625,3.375]; I2 = 17.5 %; z = 4.57; *p* < 0.0001	2.990[1.666,5.366]; I2 = 24.6 %; z = 3.67; *p* < 0.0001	2.230[1.642,3.029]; I2 = .%; z = 5.14; *p* < 0.0001
Luminal A vs. Luminal B vs. Luminal HER2 vs. HER2-enriched vs. Basal-like	_	*p* < 0.0001	_	*p* < 0.0001	*p* < 0.0001	_

*OR* odds ratio; *LN* lymph node; *ER* estrogen receptor; *PR* progesterone receptor; *HER2* human epidermal growth factor receptor-2; *TNBC* triple-negative breast cancer; “-” no results owing to insufficient studies

### Tumour size

The incidence of FOXP3+ TILs in the tumour size >2 cm group was higher than tumour size ≤2 cm group, but the difference did not reach statistical significance (OR = 1.151, 95 % CI [0.997, 1.329], I^2^ = 25.0 %). Then subgroup analysis were performed on TILs site (Intratumoural: OR = 1.098, 95 % CI [0.966, 1.247], I^2^ = 0.0 %; Total: OR = 1.268, 95 % CI [0.954, 1.686], I^2^ = 37.0 %) and different countries (Asia: OR = 1.296, 95 % CI [0.867, 1.935], I^2^ = 54.9 %; Europe and North America: OR = 1.146, 95 % CI [1.016, 1.293], I^2^ = 0.0 %). The differences were statistically significant in the European and American group.

### Histological grade

The detection of FOXP3+ TILs in histopathologic specimen may indicate the degree of histological grade (III versus I, overall: OR = 3.769, 95 % CI [2.596, 5.472], I^2^ = 64.6 %; III versus II, OR = 2.299, 95 % CI [1.719,3.075], I^2^ = 80.3 %; II versus I, OR = 1.596, 95 % CI [1.172,2.174], I^2^ = 51.3 %). Then, subgroup analyses were completed on TILs site (Intratumoural: III versus I, OR = 3.360, 95 % CI [1.774, 6.363], I^2^ = 79.0 %; III versus II, OR = 1.945, 95 % CI [1.551,2.439], I^2^ = 56.9 %; II versus I, OR = 1.790, 95 % CI [1.191,2.691], I^2^ = 51.2 %. Total: III versus I, OR = 4.298, 95 % CI [3.221, 5.736], I^2^ = 0.0 %; III versus II, OR = 3.422, 95 % CI [2.706,4.326], I^2^ = 0.0 %; II versus I, OR = 1.260, 95 % CI [0.947,1.677], I^2^ = 18.7 %.) and different countries (Asia: III versus I, OR = 6.248, 95 % CI [3.627, 10.763]; III versus II, OR = 2.287, 95 % CI [1.740,3.005]; II versus I, OR = 2.732, 95 % CI [1.636,4.562]. Europe and North America: III versus I, OR = 3.342, 95 % CI [2.270, 4.920], I^2^ = 60.0 %; III versus II, OR = 2.304, 95 % CI [1.561,3.400], I^2^ = 85.2 %; II versus I, OR = 1.351, 95 % CI [1.087, 1.680], I^2^ = 0.0 %).

### ER, PR and HER2 status

The incidence of FOXP3+ TILs was significantly different between the ER positive and ER negative groups (overall: OR = 0.435, 95 % CI [0.287, 0.660], I^2^ = 90.3 %; Intratumoural: OR = 0.571 95 % CI [0.276, 1.181], I^2^ = 95.7 %; Total: OR = 0.347, 95 % CI [0.252, 0.478], I^2^ = 31.7 %; Asia: OR = 0.419, 95 % CI [0.193, 0.908], I^2^ = 88.8 %; Europe and North America: OR = 0.481, 95 % CI [0.324, 0.714], I^2^ = 84.8 %), as well as PR positive and negative groups (overall: OR = 0.493, 95 % CI [0.296, 0.822], I^2^ = 89.9 %; Intratumoural: OR = 0.417 95 % CI [0.128, 1.357], I^2^ = 96.8 %; Total: OR = 0.501, 95 % CI [0.405, 0.621], I^2^ = 0.0 %; Asia: OR = 0.432, 95 % CI [0.195, 0.959], I^2^ = 85.1 %; Europe and North America: OR = 0.594, 95 % CI [0.373, 0.945], I^2^ = 79.2 %). Moreover, there was a significant difference in the incidence of FOXP3+ TILs detection between the HER2 positive group and HER2 negative group (overall: OR = 1.896, 95 % CI [1.335, 2.692], I^2^ = 75.1 %; Intratumoural: OR = 1.141 95 % CI [0.718, 1.814], I^2^ = 81.6 %; Total: OR = 3.651, 95 % CI [2.638, 5.052], I^2^ = 0.0 %; Asia: OR = 1.684, 95 % CI [0.881, 3.218], I^2^ = 74.4 %; Europe and North America: OR = 2.059, 95 % CI [1.203, 3.523], I^2^ = 81.0 %).

### Molecular subtypes

Those studies which included five molecular subtypes: luminal A, luminal B, luminal-HER2, HER2 enriched and basal-like showed that the status of FOXP3+ TILs infiltration was increased corresponding to the order of the molecular subtypes from well to poor. The meta-analysis of all involved studies on the five molecular subtypes showed a significant difference in the status of FOXP3+ TILs infiltration among the five molecular subtypes (*p* < 0.0001). The incidence of TNBC was more likely to increase in high FOXP3+ TILs group than in low FOXP3+ TILs group (overall: OR = 2.456, 95 % CI [1.801, 3.348], I^2^ = 11.3 %; Intratumoural: OR = 3.514 95 % CI [1.563, 7.901], I^2^ = .%; Total: OR = 2.342, 95 % CI [1.625, 3.375], I^2^ = 17.5 %; Asia: OR = 2.990, 95 % CI [1.666, 5.366], I^2^ = 24.6 %; Europe and North America: OR = 2.230, 95 % CI [1.642,3.029]).

### Impact of FOXP3+ TILs on survival outcomes (RFS and OS)

To evaluate the prognostic effect for detection of FOXP3+ TILs in breast cancer patients more deeply, a meta-analysis was performed on HR for RFS or OS. HRs for RFS were available in eight studies. The evaluated pooled HRs indicated that high FOXP3+ TILs group was associated with a significantly decreased RFS (HR = 1.752, 95 % CI [1.188–2.584], p = 0.005). As shown in the subgroup analysis based on TILs site, a poor prognosis for RFS in patients with breast cancer was shown by the detection of FOXP3+ TILs in Intratumoural and Peritumoral, but not in Total (Intratumoural: HR = 1.983, 95 % CI [1.232, 3.190], I^2^ = 44.7 %; Peritumoral: HR = 2.206, 95 % CI [1.287, 3.781], I = 0.0 %; Total: HR = 1.312, 95 % CI [0.580, 2.969], I^2^ = 79.8 %). Sensitivity analysis was completed without the low quality studies (NOS score < 5) and the results were the same (overall: HR = 1.741, 95%CI 1.114- 2.720; *P* = 0.015). But in TNBC patients, evaluated pooled HRs indicated that high FOXP3+ TILs group was associated with a significantly increased RFS (HR =0.503, 95 % CI [0.324–0.779], *p* = 0.002).

**Fig. 2:**
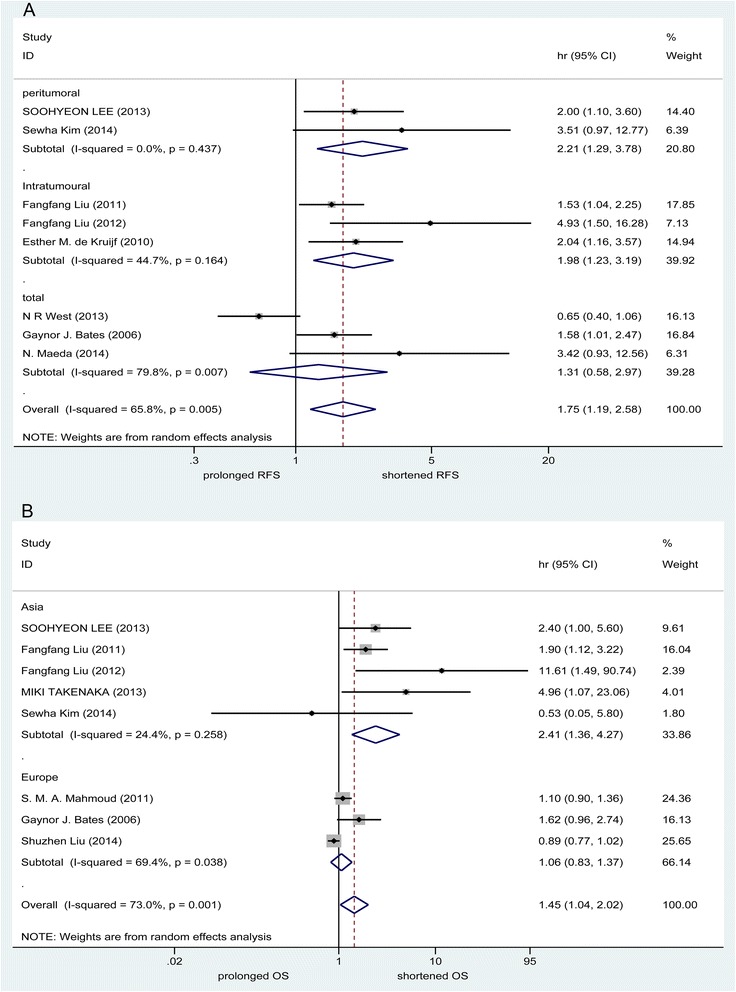
Evaluated hazard ratios (HR) summary for RFS (**a**) and OS (**b**). **a** HR for recurrence-free survival (RFS) with FOXP3+ TILs detection. **b** HR for overall survival (OS) with FOXP3+ TILs detection

**Fig. 3:**
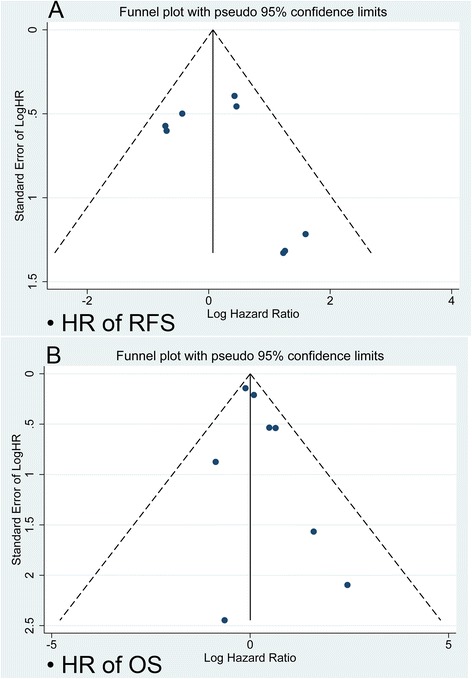
Funnel plot for potential publication bias. **a** Funnel plot analysis of studies on RFS **b** Funnel plot analysis of studies on OS. The funnel plot indicates that there was no significant publication bias

Furthermore, eight studies provided HRs on OS and the pooled results showed that breast cancer patients in the high FOXP3+ TILs group were significantly associated with a poor OS (HR =1.447, 95 % CI [1.037–2.019], *p* = 0.030). The pooled results of the subgroup analysis were similar to the results of the overall analysis in the Asia group patients (Asia: HR = 2.413, 95 % CI [1.363, 4.270], I^2^ = 24.4 %), but not in the Europe and North America group patients (Europe and North America: HR = 1.064, 95 % CI [0.827, 1.368], I^2^ = 69.4 %). On the contrary, the pooled results showed that TNBC patients in the high FOXP3+ TILs group were significantly associated with a favourable OS (HR =0.509, 95 % CI [0.356–0.728], *p* < 0.001). The evaluated pooled HRs for PFS and OS are summarized in Fig. 2. Egger’s test was used to detect publication bias. There were no significant publication bias was found (Fig. 3).

## Discussion

Although the application of standardized comprehensive treatment has been significantly improved the prognosis of breast cancer patients, but the tumor recurrence and metastasis is still a serious challenge for doctors and patients. Breast cancer is a very heterogeneous disease, which can be categorized into four main molecular subclasses based on hormone receptor and HER-2 expression. Although these subclasses have different clinical and biological characteristics, as strong heterogeneity within subgroups, such biology-based classification is still unsatisfactory. Interaction between malignant tissue and the immune system play a critical role in the process of tumor growth and metastasis. FOXP3 has been considered the most specific marker for Treg cells [16, 17]. More and more evidence indicates that regulatory T cells play an important role in immune evasion mechanisms of cancer [9–12]. However, the clinicopathological and prognostic significance of FOXP3+ TILs detection in patients with breast cancer remains controversial. This meta-analysis provided evidence to estimate the significance of FOXP3+ TILs detection in patients with breast cancer by summarizing all related studies.

Our present meta-analysis demonstrated that the detection of FOXP3+ TILs was feasible on core-needle biopsy and excisional specimen and could act as a risk factor for lymph node metastasis in patients with breast cancer. Our pooled results indicated that high levels of FOXP3+ TILs were significantly associated with high histological grade. Furthermore, our pooled analysis showed that the presence of high levels of FOXP3+ TILs was associated with ER negative, PR negativity, HER2 Positive and TNBC. This conclusion was further supported by the meta-analysis results on RFS and OS. Approximately two thirds of the patients diagnosed with invasive breast cancer have positive hormone receptors [39]. Most of the included studies reported that FOXP3+ TILs was an indicator of poor prognosis applied unstratified breast cancer. Therefore, our pooled results might largely reflect the majority ER Positive population. Subsequently, sensitivity analysis confirmed the results were still significant. No publication bias was confirmed with a funnel plot. There were several possible explanations for the correlation between FOXP3+ TILs and lymph node metastasis and poor survival. One possible explanation may be that FOXP3+ TILs reflect tumor-induced immune evasion in breast cancers. In addition, high levels of FOXP3+ TILs was associated with poor survival factors, such as high histological grade, hormone receptor negative and HER2 Positive.

But in TNBC patients, evaluated pooled HRs indicated that high FOXP3+ TILs group was associated with a significantly improved RFS and OS. So far, very few studies have been powered to evaluate if FOXP3+ TILs influence clinical outcomes in different breast cancer molecular subtypes. Therefore, this subgroup analyses still have limited power. There were several possible explanations for this result. The main explanation may be that the favorable prognostic effect of FOXP3+ TILs in TNBC breast cancer may be primarily due to CD8+ T-cell infiltration. In addition, Tregs require close contact with target cells to exert suppression [40]. Currently, one research indicates that fewer than 20 % of CD4 + FOXP3+ lymphocytes were in direct contact with CD8+ TIL in the triple negative cohort [32]. Therefore, Tregs in TNBC may not exert significant suppression on CTLs. Moreover, multiple important factors of anti-tumour immunity can be active in TNBC despite the presence of Tregs. The prognostic correlations of FOXP3+ TILs could be affected by tumor microenvironment, including tumor location, histological and molecular subtypes, as well as different types of immune response. Further studies to explore the functional status and action modes of different subsets of TILs in different breast cancer molecular subtypes will lead us to further understand the mechanisms and provide additional clues for immunotherapy.

This meta-analysis has several limitations. First, the limited number of stratified breast cancer studies would have influenced the statistical power of our results. Second, heterogeneity could not be eliminated, its existence forced us to use a relatively conservative random effect model. Third, our research is based on statistical data, rather than individual patient data, which may not be able to provide a robust estimate of association. Despite the limitations of our study, our meta-analysis is the first study to demonstrate the correlation between FOXP3+ TILs and the clinicopathological characteristics and prognosis in breast cancer.

## Conclusions

In conclusion, our meta-analysis demonstrates that the presence of high levels of FOXP3+ TILs is associated with prognosis for breast cancer patients and predicts lymph node metastasis, hormone receptor and HER-2 status. In the future, high-quality, well designed and large-scale multicenter studies are needed to explore the functional role of different TILs subsets in different breast cancer molecular subtypes. In addition, it can provide the basis for the immunotherapy of different molecular subtypes of breast cancer.

## Abbreviations

TILs: Tumor-infiltrating lymphocytes
HR: Hazard ratio
OR: Odds ratio
CI: Confidence intervals
RFS: Recurrence-free survival
OS: Overall survival
Treg cells: Regulatory T cells
IHC: Immunohistochemistry
CRT: Chemoradiotherapy
TMA: Tissue microarrays
B-NC: Before Neoadjuvant chemotherapy
post: Postoperative
NR: Not reported
pCR: Pathologic complete response
LN: Lymph node
ER: Estrogen receptor
PR: Progesterone receptor
HER2: Human epidermal growth factor receptor-2
TNBC: Triple-negative breast cancer

## References

[1] Siegel R, Naishadham D, Jemal A (2013). Cancer statistics, 2013. CA Cancer J Clin.

[2] Jemal A, Bray F, Center MM, Ferlay J, Ward E, Forman D (2011). Global cancer statistics. CA Cancer J Clin.

[3] Caras I, Grigorescu A, Stavaru C, Radu DL, Mogos I, Szegli G, Salageanu A (2004). Evidence for immune defects in breast and lung cancer patients. Cancer Immunol Immunother.

[4] Andre F, Dieci MV, Dubsky P, Sotiriou C, Curigliano G, Denkert C, Loi S (2013). Molecular pathways: involvement of immune pathways in the therapeutic response and outcome in breast cancer. Clin Cancer Res.

[5] Fridman WH, Pages F, Sautes-Fridman C, Galon J (2012). The immune contexture in human tumours: impact on clinical outcome. Nat Rev Cancer.

[6] Mahmoud SM, Paish EC, Powe DG, Macmillan RD, Grainge MJ, Lee AH, Ellis IO, Green AR (2011). Tumor-infiltrating CD8+ lymphocytes predict clinical outcome in breast cancer. J Clin Oncol.

[7] Liu F, Lang R, Zhao J, Zhang X, Pringle GA, Fan Y, Yin D, Gu F, Yao Z, Fu L (2011). CD8(+) cytotoxic T cell and FOXP3(+) regulatory T cell infiltration in relation to breast cancer survival and molecular subtypes. Breast Cancer Res Treat.

[8] Mellman I, Coukos G, Dranoff G (2011). Cancer immunotherapy comes of age. Nature.

[9] Levings MK, Sangregorio R, Roncarolo MG (2001). Human cd25(+)cd4(+) t regulatory cells suppress naive and memory T cell proliferation and can be expanded in vitro without loss of function. J Exp Med.

[10] Ng WF, Duggan PJ, Ponchel F, Matarese G, Lombardi G, Edwards AD, Isaacs JD, Lechler RI (2001). Human CD4(+)CD25(+) cells: a naturally occurring population of regulatory T cells. Blood.

[11] Shevach EM (2002). CD4+ CD25+ suppressor T cells: more questions than answers. Nat Rev Immunol.

[12] Bach JF (2003). Regulatory T cells under scrutiny. Nat Rev Immunol.

[13] Coffer PJ, Burgering BM (2004). Forkhead-box transcription factors and their role in the immune system. Nat Rev Immunol.

[14] Hori S, Nomura T, Sakaguchi S (2003). Control of regulatory T cell development by the transcription factor Foxp3. Science (New York, NY).

[15] Sakaguchi S, Ono M, Setoguchi R, Yagi H, Hori S, Fehervari Z, Shimizu J, Takahashi T, Nomura T (2006). Foxp3+ CD25+ CD4+ natural regulatory T cells in dominant self-tolerance and autoimmune disease. Immunol Rev.

[16] deLeeuw RJ, Kost SE, Kakal JA, Nelson BH (2012). The prognostic value of FoxP3+ tumor-infiltrating lymphocytes in cancer: a critical review of the literature. Clin Cancer Res.

[17] Sakaguchi S (2005). Naturally arising Foxp3-expressing CD25 + CD4+ regulatory T cells in immunological tolerance to self and non-self. Nat Immunol.

[18] Higgins J, Green S. Cochrane Handbook for Systematic Reviews of Interventions Version 5.1.0 [updated March 2011]. The Cochrane Collaboration 2011: Available from www.cochrane-handbook.org.

[19] Stang A (2010). Critical evaluation of the Newcastle-Ottawa scale for the assessment of the quality of nonrandomized studies in meta-analyses. Eur J Epidemiol.

[20] Tierney JF, Stewart LA, Ghersi D, Burdett S, Sydes MR (2007). Practical methods for incorporating summary time-to-event data into meta-analysis. Trials.

[21] Higgins JP, Thompson SG, Deeks JJ, Altman DG (2003). Measuring inconsistency in meta-analyses. BMJ.

[22] Moher D, Liberati A, Tetzlaff J, Altman DG, Group P (2009). Preferred reporting items for systematic reviews and meta-analyses: the PRISMA statement. PLoS Med.

[23] Bates GJ, Fox SB, Han C, Leek RD, Garcia JF, Harris AL, Banham AH (2006). Quantification of regulatory T cells enables the identification of high-risk breast cancer patients and those at risk of late relapse. J Clin Oncol.

[24] Aruga T, Suzuki E, Saji S, Horiguchi SI, Horiguchi K, Sekine S, Kitagawa D, Funata N, Toi M, Sugihara K (2009). A low number of tumor-infiltrating FOXP3-positive cells during primary systemic chemotherapy correlates with favorable anti-tumor response in patients with breast cancer. Oncol Rep.

[25] De Kruijf EM, Van Nes JGH, Sajet A, Tummers QRJG, Putter H, Osanto S, Speetjens FM, Smit VTHBM, Liefers GJ, Van De Velde CJH (2010). The predictive value of HLA class I tumor cell expression and presence of intratumoral tregs for chemotherapy in patients with early breast cancer. Clin Cancer Res.

[26] Ladoire S, Mignot G, Dabakuyo S, Arnould L, Apetoh L, Rebe C, Coudert B, Martin F, Bizollon MH, Vanoli A (2011). In situ immune response after neoadjuvant chemotherapy for breast cancer predicts survival. J Pathol.

[27] Mahmoud SMA, Paish EC, Powe DG, MacMillan RD, Lee AHS, Ellis IO, Green AR (2011). An evaluation of the clinical significance of FOXP3+ infiltrating cells in human breast cancer. Breast Cancer Res Treat.

[28] Liu F, Li Y, Ren M, Zhang X, Guo X, Lang R, Gu F, Fu L (2012). Peritumoral FOXP3(+) regulatory T cell is sensitive to chemotherapy while intratumoral FOXP3(+) regulatory T cell is prognostic predictor of breast cancer patients. Breast Cancer Res Treat.

[29] Kim ST, Jeong H, Woo OH, Seo JH, Kim A, Lee ES, Shin SW, Kim YH, Kim JS, Park KH (2013). Tumor-infiltrating lymphocytes, tumor characteristics, and recurrence in patients with early breast cancer. Am J Clin Oncol.

[30] Lee S, Cho EY, Park YH, Ahn JS, Im YH (2013). Prognostic impact of FOXP3 expression in triple-negative breast cancer. Acta Oncologica (Stockholm, Sweden).

[31] Takenaka M, Seki N, Toh U, Hattori S, Kawahara A, Yamaguchi T, Koura K, Takahashi R, Otsuka H, Takahashi H (2013). FOXP3 expression in tumor cells and tumor-infiltrating lymphocytes is associated with breast cancer prognosis. Molecular andClinical Oncology.

[32] West NR, Kost SE, Martin SD, Milne K, Deleeuw RJ, Nelson BH, Watson PH (2013). Tumour-infiltrating FOXP3(+) lymphocytes are associated with cytotoxic immune responses and good clinical outcome in oestrogen receptor-negative breast cancer. Br J Cancer.

[33] Kim S, Lee A, Lim W, Park S, Cho MS, Koo H, Moon BI, Sung SH (2014). Zonal difference and prognostic significance of Foxp3 regulatory T cell infiltration in breast cancer. J Breast Cancer.

[34] Liu S, Foulkes WD, Leung S, Gao D, Lau S, Kos Z, Nielsen TO. Prognostic significance of FOXP3+ tumor-infiltrating lymphocytes in breast cancer depends on estrogen receptor and human epidermal growth factor receptor-2 expression status and concurrent cytotoxic T-cell infiltration. Breast Cancer Research. 2014, 16(5). 10.1186/s13058-014-0432-8 PMC4303113 25193543

[35] Maeda N, Yoshimura K, Yamamoto S, Kuramasu A, Inoue M, Suzuki N, Watanabe Y, Maeda Y, Kamei R, Tsunedomi R (2014). Expression of B7-H3, a potential factor of tumor immune evasion in combination with the number of regulatory T cells, affects against recurrence-free survival in breast cancer patients. Ann Surg Oncol.

[36] Sun S, Fei X, Mao Y, Wang X, Garfield DH, Huang O, Wang J, Yuan F, Sun L, Yu Q (2014). PD-1(+) immune cell infiltration inversely correlates with survival of operable breast cancer patients. Cancer Immunol Immunother.

[37] Tsang JY, Hui SW, Ni YB, Chan SK, Yamaguchi R, Kwong A, Law BK, Tse GM (2014). Lymphocytic infiltrate is associated with favorable biomarkers profile in HER2-overexpressing breast cancers and adverse biomarker profile in ER-positive breast cancers. Breast Cancer Res Treat.

[38] Seo AN, Lee HJ, Kim EJ, Kim HJ, Jang MH, Lee HE, Kim YJ, Kim JH, Park SY (2013). Tumour-infiltrating CD8+ lymphocytes as an independent predictive factor for pathological complete response to primary systemic therapy in breast cancer. Br J Cancer.

[39] Baselga J, Campone M, Piccart M, Burris HA, Rugo HS, Sahmoud T, Noguchi S, Gnant M, Pritchard KI, Lebrun F (2012). Everolimus in postmenopausal hormone-receptor-positive advanced breast cancer. N Engl J Med.

[40] Shevach EM (2009). Mechanisms of foxp3+ T regulatory cell-mediated suppression. Immunity.

